# Supplementary material for: Ferroptosis Inducers Erastin and RSL3 Enhance Adriamycin and Topotecan Sensitivity in ABCB1/ABCG2-Expressing Tumor Cells
Source: Int J Mol Sci. 2025 Jan 14;26(2):635. doi: 10.3390/ijms26020635 (PMC11765678; doi:10.3390/ijms26020635)
Supplement: Supplementary file 1 [file ijms-26-00635-s001.zip › ijms-3382052-supplementary.pdf]

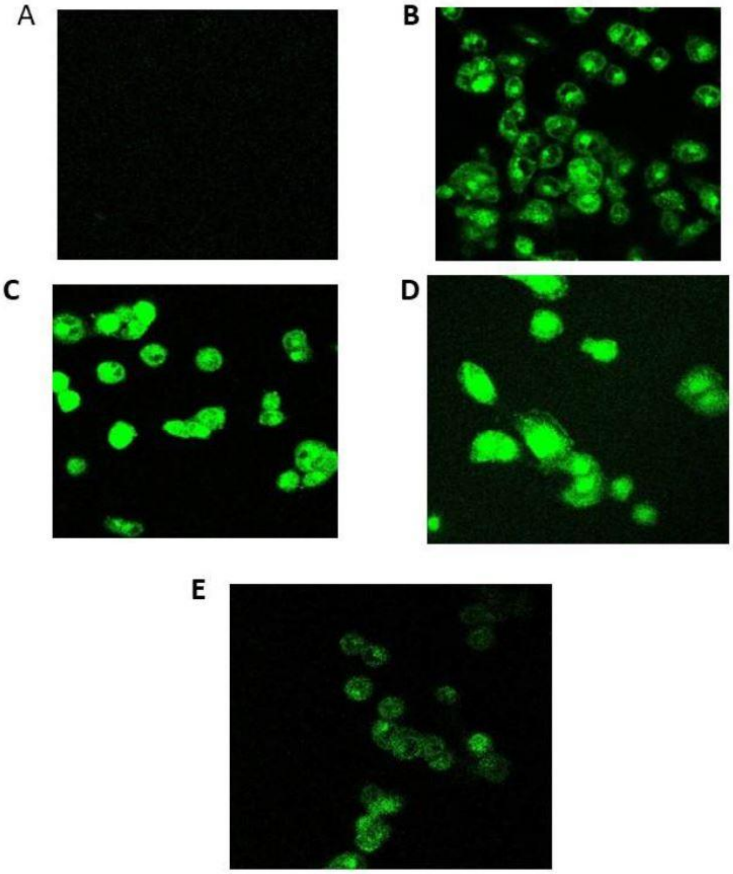

Supplementary Figure-1: The confocal microscopy studies for the uptake of Adriamycin with NCI/ADR-RES (R) cells (A) control, (B) in the presence of 5 μM Adriamycin, (C) in the presence of 5 μM Adriamycin and Erastin, (D) in the presence of Adriamycin and10 μM verapamil and (E) in the presence of 5 μM Erastin and 10 μM 1400W. A representative scan is shown here. The fluorescence intensity was quantitated from total of at least three separate cell scans and data in Figure-4 represent total of at least 3 separate experiments.

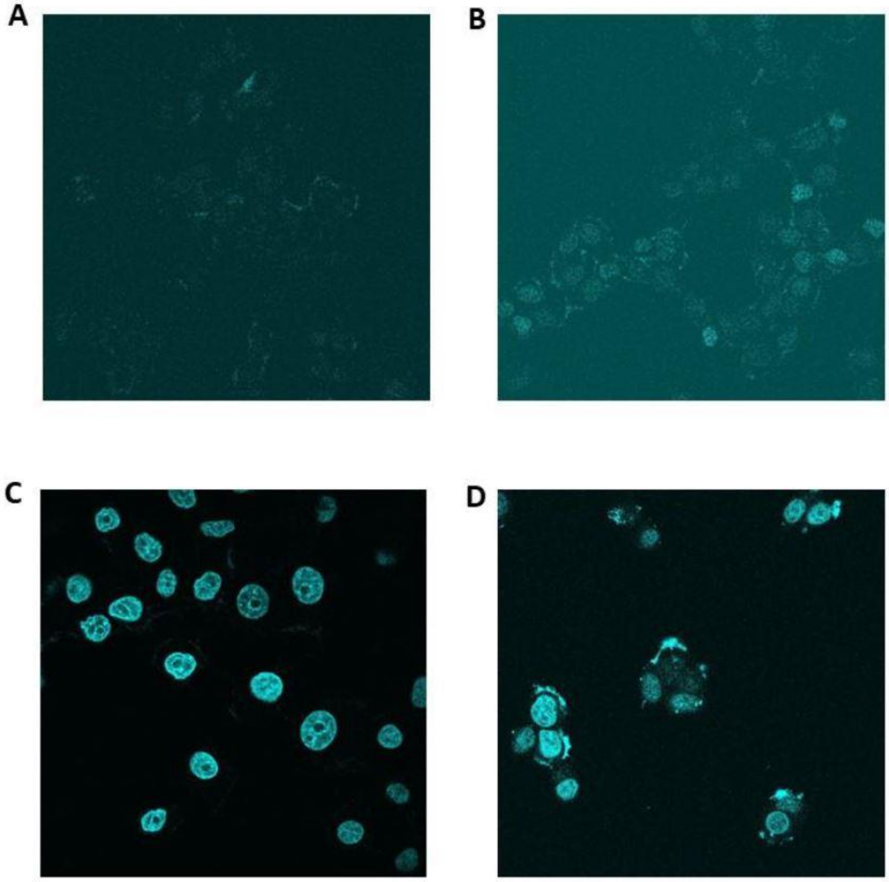

Supplementary Figure-2: The confocal microscopy studies for the uptake of Hoechst dye with MXR cells, (A) control, (B) in the presence of 5 μM Erastin, (C) in the presence of 10 μM Ko143 and (D) in the presence of 5 μM Erastin and 10 μM 1400W. A representative scan is shown here. The fluorescence intensity was quantitated from at least three separate cell scans and data in Figure-5 represent total of at least 3 separate experiments.

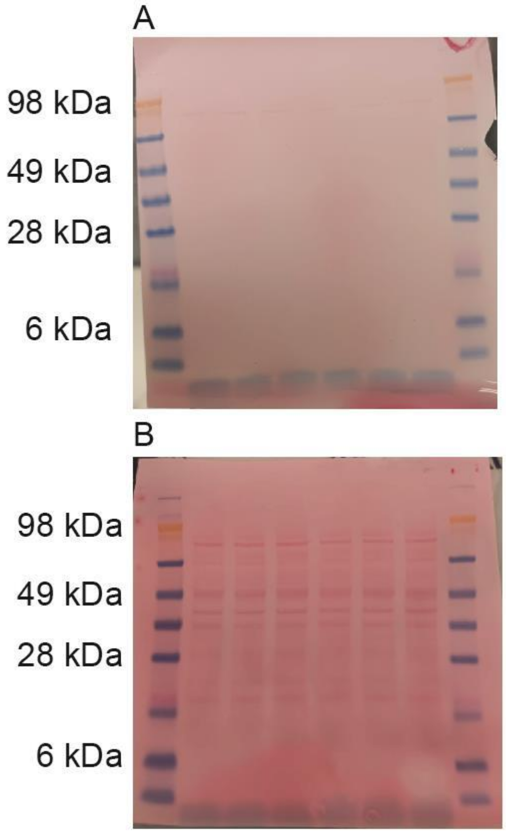

Supplementary Figure 3: Ponceau staining for total protein for (A) BCRP in MCF-7/MXR and MCF-7 cells and (B) P-170 in OVCAR-8 and NCI/ADR-RES cells.
